# Title TBA: Revising the Abstract Submission Process

**DOI:** 10.1016/j.tics.2018.01.008

**Published:** 2018-04

**Authors:** Roni Tibon, CBU Open Science Committee, Richard Henson

**Affiliations:** 1 MRC Cognition and Brain Sciences Unit, University of Cambridge, Cambridge, UK

**Keywords:** academic conferences, Open Science, preregistration

## Abstract

Academic conferences are among the most prolific scientific activities, yet the current abstract submission and review process has serious limitations. We propose a revised process that would address these limitations, achieve some of the aims of Open Science, and stimulate discussion throughout the entire lifecycle of the scientific work.

Scientific conferences provide opportunities to share scientific results, learn about recent advances in the field, and establish networks and collaborations. For many, especially early career researchers, travel funding is available only when a work (talk or poster) is presented. However, most conferences require the submission of an abstract that describes completed work many months before the conference itself. This has at least two drawbacks. First, allowing only completed work means that researchers cannot receive feedback on prior stages, when such feedback is arguably most valuable. Second, it encourages researchers to submit rushed and potentially premature analyses. In this Opinion article, we critique the typical abstract submission process for posters in the field of cognitive neuroscience (although it is likely to apply to other fields, too) and propose a revised submission and review process that addresses these problems and adheres to the principles of Open Science [1].

## Getting Your Abstract Accepted

For the major cognitive neuroscience conferences, researchers need to submit their abstracts approximately 5 months before the conference (Table 1). Many of these conferences specifically require that the abstract describes a completed work. For example, the instructions for the 25th Annual Meeting of the Cognitive Neuroscience Society (CNS) state

‘Your abstract must contain the specific goals of the study, the methods used, a summary of the results, and a conclusion. DO NOT SUBMIT AN ABSTRACT FOR PLANNED WORK. THE ABSTRACT MUST ENTAIL DATA ANALYSES AND RESULTS’

**Table 1:** Ten Examples of Major Cognitive Neuroscience Conferences in 2017–2018, the Deadline for Abstract Submission (for Poster Presentations), the Start Day of the Conference, and the Time Elapsed between These Two Dates

Conference	Submission deadline	Conference start date	Time elapsed (months)
39th Annual Meeting of the Cognitive Science Society (CogSci)	1 February 2017	26 July 2017	6
13th International Conference of Cognitive Neuroscience (ICON)	31 March 2017	5 August 2017	4.5
20th Conference of the European Society for Cognitive Psychology (ESCoP)	30 April 2017	3 September 2017	4
57th Annual Meeting of the Society for Psychophysiological Research (SPR)	3 April 2017	11 October 2017	6
Psychonomic Society’s 58th Annual Meeting	1 June 2017	9 November 2017	5
Society of Neuroscience’s 47th Annual Meeting	4 May 2017	11 November 2017	6
25th Annual Meeting of the CNS	1 November 2017	24 March 2018	4.5
OHBM 2018 Annual Meeting	15 December 2017	17 June 2018	6
30th Association for Psychological Science (APS) Annual Convention	31 January 2018	24 May 2018	4
11th FENS Forum of Neuroscience	13 February 2018	07 July 2018	5
		Average (SD):	5.1 (0.8)

while those for the 2018 Annual Meeting of the Organization of Human Brain Mapping (OHBM) state

‘The abstract should describe only work (experiments and analysis) that has already been completed, not work that is planned for the interval between abstract submission and the conference.’

Once submitted, abstracts are peer reviewed by a selection committee. Normally, each member scores a portion of the abstracts and each abstract is scored by more than one member. Assessment criteria are normally predefined (although more transparently for some conferences than others). Abstracts that receive the highest scores are accepted and authors are notified of acceptance 2–3 months before the conference. When attending the conference, it is expected that the work presented will match the abstract.

## Works Fine! What’s Wrong?

Suppose that a well-designed experiment is at a relatively advanced stage where all the data are collected but not yet analysed. When abstract submission is due, the researchers are faced with several options. First, they can decide not to submit an abstract to this conference and instead wait for a different conference with a later deadline. By then, they would be able to present a completed work that might already be a preprint on an archive and possibly in press or even in print with a journal. While this might still be used as a ‘ticket’ to attend the conference, the benefits of the conference for the authors would be reduced because the opportunity to gain feedback on how to improve analyses or test alternative hypotheses has largely passed. Furthermore, colleagues with shared scientific interests would not become aware of the study until it is too late for collaborations. Indeed, if every author were to submit work that is already in the public domain, the value of poster sessions at large international conferences becomes unclear.

A second option is to quickly analyse the data to be able to include preliminary, and often premature, results. However, such results are likely to change by the time of the conference, leaving the researcher uncomfortable and the conference program outdated. Mismatches between abstracts and the content of posters are usually overlooked by conference organizers, such that the abstracts in these programs *are* often outdated. Accordingly, the programs are rarely used as a reliable source of information regarding scientific results and usually only serve to browse for keywords or topics of interests.

Thus, in reality, work in progress is rarely eligible for a conference − but what about even earlier stages in the scientific process? These include ideas for hypotheses and experimental designs that have not been executed yet, where feedback can help to determine whether they need refinement or extra controls or are not worth the effort. One deceptive possibility is to submit an abstract for work that is not yet undertaken but pretend that it has been (e.g., by adding bland statements describing the results). Should a work at this stage be considered for a poster presentation in a conference in its own right? We think the answer is ‘yes’. Bouncing ideas off colleagues is an essential means for testing their validity, feasibility, consistency, and merit. This preliminary discussion can make science more efficient, by not wasting time on work that is unlikely to be valued by the community and by encouraging collaboration when two groups plan similar studies. Some conferences – such as the Psychology, Health, and Medicine Conference [2] – have realised the importance of discussion at early stages and started to permit submission of posters outlining research in the planning phase. Nevertheless, the vast majority of conferences still require presentation of completed research. In these conferences some of the discussion of planned research no doubt takes place informally, but our point is that it could be encouraged and formalised by widening the scope of abstracts. Similar changes have already been formalised in the peer review of preregistration reports 3, 4, 5, 6, 7; abstracts for planned work can serve some of the functions of preregistration reports, as we expand below.

## So Let’s Get Practical

### A Revised Submission Process

We propose that abstracts for poster presentations could be submitted under three ‘progression stages’: (i) planned studies – studies that are only planned at the time of submission, and may be refined by the time of the conference, but are not yet executed; (ii) collected data – studies for which data have been collected by the time of submission and may be partially/fully analysed by the time of the conference; and (iii) analysed data – studies that are partially/fully analysed at the time of submission and will be fully analysed by the time of the conference.

**Figure 1:**
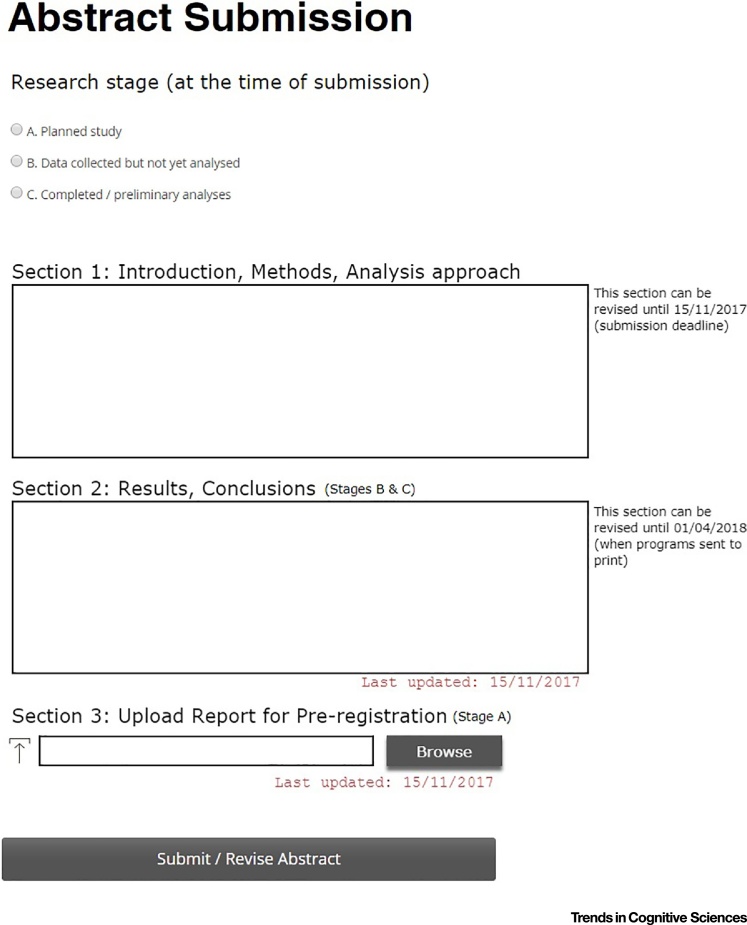
An Example of an Abstract Submission Page That Would Support the Proposed Process.

The submission form could contain three sections (Figure 1). In Section 1 authors describe the theoretical background (including motivation and predictions) of the study, the experimental methods, and the analysis approach that was, or will be, used. Once the submission deadline has passed, this section cannot be revised. This section will be mandatory for all progression stages, which then differ in how they handle the other sections.

Stage A. At the time of submission, authors complete Section 1 only. After receiving an acceptance notification, they will be asked to submit a full preregistration report (Section 3). The deadline for uploading the report will be just before the conference. Until the report is submitted, the status of the abstract is ‘accepted – pending report submission’.

Stage B. At the time of submission, authors complete Section 1 and are able (but not required) to provide some results in Section 2 (e.g., initial quality assurance). Following acceptance authors complete Section 2, describing the results and conclusions of the study. The deadline for completing this section would be just before the conference. Until Section 2 is complete, the status of the abstract is ‘accepted – pending results submission’.

Stage C. At the time of submission, authors complete Sections 1 and 2. Nevertheless, they will be able to revise the information provided in Section 2 (but not in Section 1) repeatedly until the time of the conference.

### The Supporting Review Process

Before the call for abstract submission, the program committee decides on scoring criteria for the review process. Scoring criteria will vary for the different progression stages. For Stages A and B, assessment will be based on Section 1, focusing on theoretical merit of the study and its predictions as well as the suitability of the analysis approach. For Stage C scoring will also include Section 2, assessing the results and the validity of the conclusions. To accommodate works in various progression stages, a predefined acceptance quota can be assigned to each stage (e.g., 20% planned studies, 30% collected data, 50% preliminarily/fully analysed data). Scoring criteria and quotas should be published online, available to authors at the time of submission.

The program committee will further nominate a poster committee who peer-review the submissions using the prespecified scoring criteria, with each abstract reviewed by at least two committee members. Because the number of submissions is likely to increase, the committee may need to be open to more members, including early career scientists who might benefit most from exposure to recent advances and from being acknowledged for their contribution (one option, adopted by the CNS, is to approach first authors of posters presented in the previous year). All abstracts will be reviewed on submission. Thus, although some of the abstracts’ sections can be revised later, these changes would not affect the acceptance decision. The last opportunity to revise Sections 2 and 3 will be just before the conference, at a date that would afford enough time to finalise the program. The finalised program will therefore include the most recent findings – those that will actually be presented at the conference.

## And Then What?

After the conference authors could load the full version of their posters to the conference website. Importantly, authors who submitted their posters under Stage A (planned research) might be able to use their revised report (amended following conference feedback) to register their work in a journal 3, 4, 6. Ideally, organisations holding the conference would use this initial ‘conference screening’ to accelerate preregistration and publication in their peer-reviewed journals.

To summarise, we believe that by revising the abstract submission and review process, conferences will be able to stimulate discussion throughout the entire lifecycle of scientific work, encourage preregistration, and foster collaborations, thereby promoting the core principles of Open Science.
